# The Cheese Production Facility Microbiome Exhibits Temporal and Spatial Variability

**DOI:** 10.3389/fmicb.2021.644828

**Published:** 2021-03-09

**Authors:** Jared Johnson, Chris Curtin, Joy Waite-Cusic

**Affiliations:** Department of Food Science and Technology, Oregon State University, Corvallis, OR, United States

**Keywords:** facility microbiome, cheese, built environment, fermentation, food

## Abstract

A primary goal of modern cheese manufacturing is consistent product quality. One aspect of product quality that remains poorly understood is the variability of microbial subpopulations due to temporal or facility changes within cheese production environments. Therefore, our aim was to quantify this variability by measuring day-day and facility-facility changes in the cheese facility microbiome. In-process product (i.e., milk and cheese) and food-contact surfaces were sampled over the course of three production days at three cheese manufacturing facilities. Microbial communities were characterized using 16S rRNA metabarcoding and by plating on selective growth media. Each facility produced near-identical Cheddar cheese recipes on near-identical processing equipment during the time of sampling. Each facility also used a common pool of *Lactococcus* starter cultures which were rotated daily as groups of 4–5 strains and selected independently at each facility. Diversity analysis revealed significant facility-facility and day-day differences at each sample location. Facility differences were greatest on the food contact surfaces (i.e., draining-matting conveyor belts), explaining between 25 and 41% of the variance. Conversely, daily differences within each facility explained a greater proportion of the variance in the milk (20% vs. 12%) and cheese (29% vs. 20%). Further investigation into the sources of these differences revealed the involvement of several industrially relevant bacteria, including lactobacilli, which play a central role in flavor and texture development during Cheddar cheese ripening. Additionally, *Streptococcus* was found to contribute notably to differences observed in milk samples, whereas *Acinetobacter*, *Streptococcus*, *Lactococcus*, *Exiguobacterium*, and Enterobacteriaceae contributed notably to differences on the food contact surfaces. Facility differences in the cheese were overwhelmingly attributed to the rotation of *Lactococcus* starter cultures, thus highlighting circumstances where daily microbial shifts could be misinterpreted and emphasizing the importance of repeated sampling over time. The outcomes of this work highlight the complexity of the cheese facility microbiome and demonstrate daily and facility-facility microbial variations which might impact cheese product quality.

## Introduction

The industrialization of food production has required that modern food manufacturing practices be highly regimented in order to create consistent products that meet consumer expectations. Despite this highly controlled approach, food manufacturers still face challenges in producing products with consistent quality, especially when producing the same product at multiple facilities. Food quality is complex and can be impacted by a combination of factors including raw ingredient heterogeneity and process changes (Gram et al., 2002). The impact of microbial populations and their dynamics in the food processing environment remain understudied.

Microbial variability in the production environment is particularly important for fermented foods, which rely on the action of microorganisms for their production. Many modern fermentation practices employ starter cultures as a means of standardizing the fermentation process; however, non-starter bacteria, which enter the food by way of the raw ingredients or the food processing environment, can also participate during fermentation and are sometimes attributed with quality changes in the finished product (Gram et al., 2002; Kandasamy et al., 2018). A prime example being Cheddar cheese production, where non-starter lactic acid bacteria (NSLAB) originating from the milk and the cheesemaking environment are sometimes associated with positive or negative quality outcomes that primarily occur during cheese ripening (Fox et al., 2017; Blaya et al., 2018).

Though decades of research have focused on identifying the microorganisms that cause these quality changes in cheese and other fermented foods, our understanding of how microbial communities vary in the production environment remains limited (Gram et al., 2002; Petruzzi et al., 2017). High throughput sequencing has opened the door to the exploration of microbial variability at the community-level and thus has allowed for initial investigations into these complex but fundamental questions. Bokulich and Mills (2013) were among the first to explore the cheese facility microbiome and revealed a complex and diverse microbial landscape that was, in large part, shaped by the environmental conditions at each processing stage (e.g., milk handling, curd processing, packaging, etc.). Despite the influence of processing stage, underlying facility-facility differences were still observed by Bokulich and Mills (2013). This led the authors to postulate that cheese facilities can harbor facility-specific microbiomes which potentially influence product quality.

Farm-level differences have likewise been observed in the milk microbiome, further supporting the idea that dairy processors can harbor site-specific microbial communities (Kable et al., 2016; Skeie et al., 2019). However, the milk microbiome can also exhibit considerable temporal variation, changing both seasonally and throughout the production day during milk processing (Kable et al., 2016, 2019; Skeie et al., 2019). Given the close relationship between milk and cheese production, it is anticipated that microbial communities would likewise change over time in the cheesemaking environment. Thus, our aim was to evaluate temporal and spatial variation of bacterial communities in the Cheddar cheese production environment by performing 16S rRNA metabarcoding and enumeration on selective growth media of samples from in-process product (i.e., milk and cheese) and swabs of food contact surfaces from three manufacturing facilities over the course of three consecutive production days. Our results show that there is significant temporal and spatial variation in these communities, and that comprehensive sampling is required to accurately characterize a cheese facility microbiome.

## Methods

### Commercial Facilities

Three cheese processing facilities (A, B, C) served as the testing sites for this research. Each facility produces more than 25,000 tons of cheese per year and produces Cheddar cheese using identical formulations and processes, on nearly identical equipment. All three facilities follow a similar production schedule—i.e., 21-h production shift with a midday wash (caustic, acid, and water rinse between the milk balance tank and the DMC) followed by a 3-h sanitation shift. These facilities source raw milk from dairy farms within 250 miles (402 km) of each facility. These facilities also produce other types of cheeses (other Cheddar recipes, other semi-hard cheeses); therefore, sampling visits were coordinated to ensure that comparable Cheddar cheese recipes were being produced at all three facilities for three consecutive production days (September 10–12, 2019). Cheddar cheese was not produced at Facility C on the first day of sampling; therefore, only two production days (days 2 and 3) were sampled at Facility C.

### Starter Rotations

**FIGURE 1:**
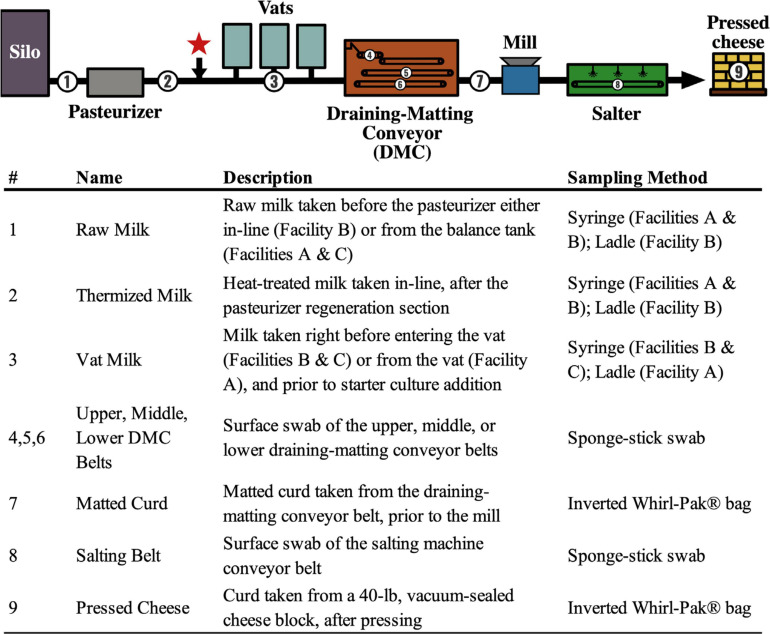
Flow diagram showing the generic Cheddar cheesemaking process and sample collection points. Numbers denote sampling locations within the cheese production facilities (A, B, C). Descriptions of each sample location and the method used are listed. The red star indicates the approximate location of starter addition. Thermization conditions used at each facility are 67–70°C for 26–28s.

Each of the participating facilities rotate 6 blends of *Lactococcus* starter cultures, each consisting of 4–5 *Lactococcus lactis* subsp. *cremoris* strains. A *Lactobacillus rhamnosus* adjunct starter is also used at each facility but is not rotated. *Lactococcus* starters are inoculated at ∼10^6^ CFU/mL via pH adjusted batch cultures injected directly into the milk-to-vat line, whereas the *Lactobacillus* starter is inoculated at ∼10^6^ CFU/mL but using a direct vat inoculation (Figure 1). *Lactococcus* blends are rotated every 24–48 h at each facility. Facilities B and C use the same *Lactococcus* starter blends, whereas half of the starter blends used at Facility A are unique. Blends used during the 3-day sampling period are shown in Supplementary Figure 1.

### Sample Collection and Processing

A total of 288 samples were collected from cheese facilities A, B, and C during three consecutive production days. All samples were collected within 15–21 h from the start of production at the sampling locations listed in Figure 1. Milk samples (50 mL) were collected from a sanitary port using a sterile syringe or from balance tanks or vats using a sanitized stainless-steel ladle. Food contact surfaces (i.e., conveyor belts) were sampled using sponge-stick swabs stored in neutralizing buffer (Solar Biologicals, Newark, DE). Curd samples (approximately 150 g) were collected directly into an inverted Whirl-Pak^®^ bag (Nasco, Fort Atkinson, WI). All samples were immediately stored on ice and shipped to Oregon State University within 36 h of collection. Samples were collected repeatedly from each location every 3 min over a 12-min period (*n* = 4). The continuous nature of the cheesemaking process means that each repeated sample represents a new section of the in-process product or processing environment. For this reason, repeated samples taken over the 12-min period were treated as biological replicates.

Upon arrival at OSU, liquid samples (milk and swab solution) were vortexed, and a 1.8 mL aliquot was taken for DNA extraction. Curd samples (10 g) were homogenized by stomaching in 90 mL of 1% sodium citrate buffer (pH 5.2). Aliquots were centrifuged at 9,500 × g for 2 min, the supernatant was removed, and the resulting pellets were stored at −80°C for later DNA extraction.

### Bacterial Enumeration and Isolation

A subset of samples (*n* = 72) was enumerated by standard serial dilution and spread plating on selective media: de Man, Rogosa and Sharpe agar [MRS; generic lactic acid bacteria (LAB)] (Neogen), Rogosa SL agar (SL; non-*Lactococcus* LAB) (HiMedia, Mumbai, India), m-Enterococcus agar (m-EA; presumptive enterococci/streptococci) (Neogen), and MacConkey agar (MAC; coliforms) (Neogen). This subset included one of the four replicate samples taken from each sampling location on each day from each facility. MRS and SL plates were incubated at 35°C for 5 days under microaerophilic conditions (5% O_2_, 10% CO_2_) in a hypoxic chamber (Bactrox, Sheldon Manufacturing, Inc., Cornelius, OR). MAC and m-EA plates were incubated at 37°C for 24 or 48 h, respectively.

Representative isolates of unique colony morphologies from MAC (*n* = 34 isolates) and m-EA (*n* = 17 isolates) plates from the draining matting conveyor (DMC) swab samples (locations 4, 5, and 6; Table 1) were streaked for isolation on tryptic soy agar supplemented with 0.6% yeast extract (TSAYE) and incubated under the same conditions as the original source media. Isolates were cultured in tryptic soy broth supplemented with 0.6% yeast extract (TSBYE) for 48 h and diluted 1:1 in 50% glycerol for long-term storage at −80°C.

**TABLE 1:** *P*-values from the Kruskal-Wallis test for differences in Shannon’s alpha diversity between facilities and between days within each facility.

Sample location	Facility	Day (Facility A)	Day (Facility B)	Day (Facility C)
Raw milk	ns	ns	ns	ns
Thermized milk	ns	ns	0.021	ns
Vat milk	0.018	0.039	ns	0.021
Upper DMC belt	<0.001	0.024	0.024	0.021
Middle DMC belt	<0.001	0.012	ns	0.021
Lower DMC belt	<0.001	0.007	0.012	ns
Salting belt	0.023	0.007	0.007	ns
Matted curd	0.002	0.007	0.023	ns
Pressed cheese	0.018	0.007	0.023	0.021

### Isolate Identification by 16S rRNA Sequencing

Bacterial isolates were identified by 16S rRNA sequencing. DNA was extracted from pure cultures of each isolate using either a crude extraction (boil in water for 10 min) or the DNeasy Blood and Tissue Kit (Qiagen, Carlsbad, CA). PCR was performed in 25 μL reactions using Platinum Hot-Start Master Mix (Thermo Fisher, Waltham, MA) and 0.2 μM the 27F/1492R universal primers (Frank et al., 2008). PCR conditions were as follows: initial denaturation at 95°C for 5 min, followed by 30 cycles of 95°C for 30 s, 51°C for 30 s, and 72°C for 2 min, and a final extension at 72°C for 10 min. PCR quality and fragment size were verified by gel electrophoresis (1% agarose gel, 10 V/cm). PCR products were cleaned with the Gel/PCR DNA fragment extraction kit (IBI Scientific, Dubuque, IA) and the DNA concentration was quantified using the Qubit 4 (Invitrogen, Carslbad, CA). Cleaned PCR products were sequenced using both 27F and 1492R primers on an ABI 3730 capillary sequencer (Thermo Fisher) by the Oregon State University Center for Genome Research and Biocomputing (CGRB, Corvallis, OR). Consensus sequences were generated from the forward and reverse sequences for each isolate using SeqTrace (Stucky, 2012). Taxonomy was assigned using the EZBioCloud Database (Yoon et al., 2017).

### DNA Extraction and High Throughput Sequencing

DNA was extracted from previously frozen milk, swab, and curd subsamples (*n* = 288) using the PowerFood Microbial DNA Isolation Kit (Qiagen, Carlsbad, CA) following manufacturer’s instructions. A single thermized milk sample from Facility C was lost due to human error during this process. Subsamples were homogenized with the Bead Ruptor 24 (Omni, Kennesaw, GA) using 10 cycles of a 15 s pulse at 8 m/s with a 55 s rest between cycles. DNA extractions and PCR reactions were validated using the ZymoBIOMICS Microbial Community Standard and Microbial Community DNA Standard (Zymo Research Corp., Irvine, CA). Non-template controls were also included on each PCR reaction plate.

PCR libraries for 16S rRNA metabarcoding were prepared in the manner described by Comeau et al. (2017) with modifications. Indexed libraries of the milk, belt swab, and cheese samples (*n* = 287) were prepared using pairwise combinations of 515F and 926R fusion primers containing Illumina i5 and i7 indices and P5 and P7 sequence adapters (Supplementary Table 1). Duplicate PCR reactions were performed using Platinum Hot-Start Master Mix. PCR conditions were as follows: initial denaturation at 94°C for 3 min, followed by 30 cycles of 94°C for 45 s, 55°C for 45 s, and 72°C for 90 s, and a final extension at 72°C for 10 min. PCR product quality and fragment size were verified by gel electrophoresis (1% agarose gel, 10 V/cm) and then normalized using SequalPrep 96-well plates (Applied Biosystems, Foster City, CA). Amplicon sequencing was carried out in two sequencing runs on a Miseq 3000 (Illumina, San Diego, CA) with 2 × 300 bp v3 chemistry at the CGRB.

### Sequence Processing

High throughput sequence data were processed using workflows in QIIME2 v2019.1.0 (Bolyen et al., 2019) and R v4.0.0 (R Core Team, 2020). Initial processing began with demultiplexed sequence files from combined sequence runs. Residual forward and reverse primer sequences were removed from the demultiplexed sequences using the QIIME2 cut-adapt plug-in (Martin, 2011). Reads were trimmed to 250 nt (forward) and 220 nt (reverse), quality filtered, merged, and chimeras were removed using the QIIME2 DADA2 plug-in (Callahan et al., 2016). The average merged contig length was 373 bp. Sequence taxonomy was assigned twice, both times using a Naïve-Bayes trained QIIME2 classifier trained to the 16S rRNA 515F/926R region using the Greengenes 99% OTU database (downloaded January 28, 2020). The first assignment of taxonomy was used for the identification and removal of sequences identified as “mitochondrial” or “chloroplast” DNA using the “filter-table” and “filter-seq” options of the QIIME2 taxa plug-in. Taxonomy was then reassigned to the resulting dataset and used for all subsequent processing in R.

QIIME2 artifacts were loaded into R using the qiime2R v0.99.23 (Bisanz, 2018) and phyloseq v1.32.0 (McMurdie and Holmes, 2013) packages. Suspected DNA contaminants were removed using the “prevalence” method in decontam v1.8.0 (Davis et al., 2018) with threshold (P^∗^) 0.495. The resulting “decontaminated” sequence files were used for all downstream analysis.

### Data Analysis

High throughput sequence data and bacterial enumeration data were analyzed in R. Rarefaction curves were produced using the “ggrare” function in ranacapa v0.1.0 (Kandlikar et al., 2018). Species richness and Shannon’s alpha diversity were estimated from rarefied abundance tables using the “estimate_richness” function in vegan v2.5.6 (Oksanen et al., 2019). Beta diversity was estimated following a center log-ratio (clr) transformation. Principle component analysis was performed on clr transformed data using the “prcomp” function in stats v3.6.2 (R Core Team, 2020). Group centroids were determined by taking the average component scores for PC1 and PC2. Individual contributions of SVs to the PC1 and PC2 variance were determined by squaring the loadings generated by prcomp. Significant differences in beta diversity between facilities and between production days nested within each facility were determined by PERMANOVA based on the Aitchison’s distance, using the “adonis2” function in vegan (formula = ∼facility^∗^day). Heterogeneity of within-group dispersion for facility and day-per-facility was determined by PERMDISP using the “betadisper” function in vegan. Core microbial species were determined using the microbiome v1.10.0 package (Lahti and Shetty, 2019) with threshold 0.90. Figures and tables were generated in R and Microsoft Excel.

## Results

### Sequencing Results and Alpha Diversity

Microbial diversity was investigated at each Cheddar cheese production facility using 16S rRNA metabarcoding. Large differences in sequencing depth were observed between sample types (i.e., milk, belts, cheese). Consequently, each sample type was rarefied to different depths accordingly prior to analysis of alpha diversity (Supplementary Figure 2).

**FIGURE 2:**
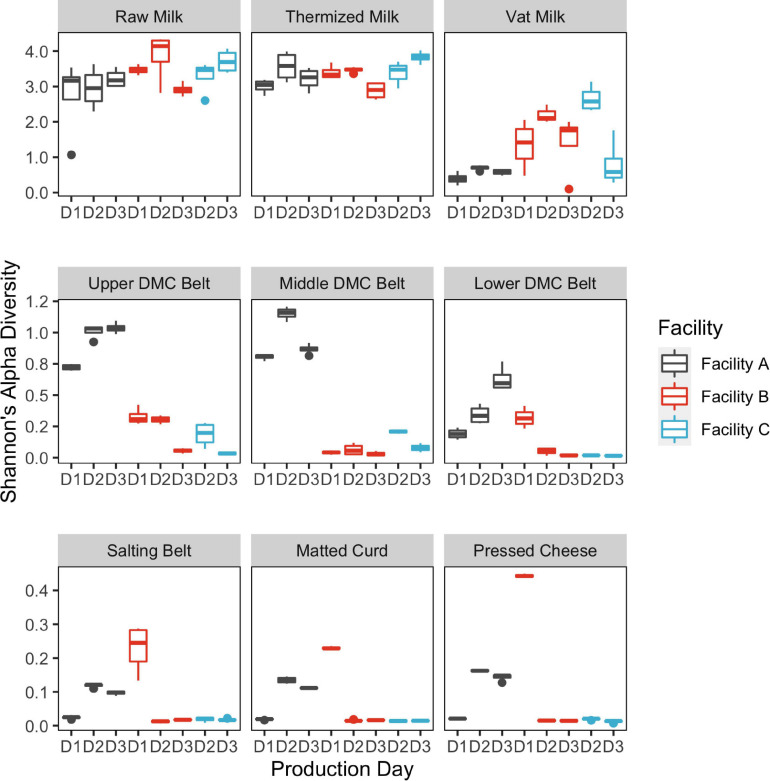
Box plots showing Shannon’s alpha diversity estimates by sample location. Bars are colored by cheesemaking facility. Repeat sampling days within each facility are displayed across the *x*-axis (*n* = 3 Facilities A and B; *n* = 2 Facility C). Decline in alpha diversity in samples collected post-thermization is partially explained by starter addition which occurs in the vat.

Alpha diversity was greatest in the milk samples and generally declined through the cheesemaking process (Figure 2). Median species richness for milk, belt, and cheese samples were 56, 8, and 6.5 sequence variants (SVs) per sample, respectively. Residual starter from previous cheese makes in the milk-to-vat line reduced species richness from a median value of 66 SVs per sample in the thermized milk to 22 SVs per sample in the vat milk. Significant differences in alpha diversity were observed between facilities at all sample locations, with the exception of thermized and raw milk samples, and between days in at least one facility for all sample locations, except raw milk (Figure 2 and Table 1). Alpha diversity at Facility A was generally greater in sample locations where significant facility differences were observed (i.e., DMC belts, salting belt, matted curd, and pressed cheese).

### Comparing Between Samples (Beta Diversity)

**FIGURE 3:**
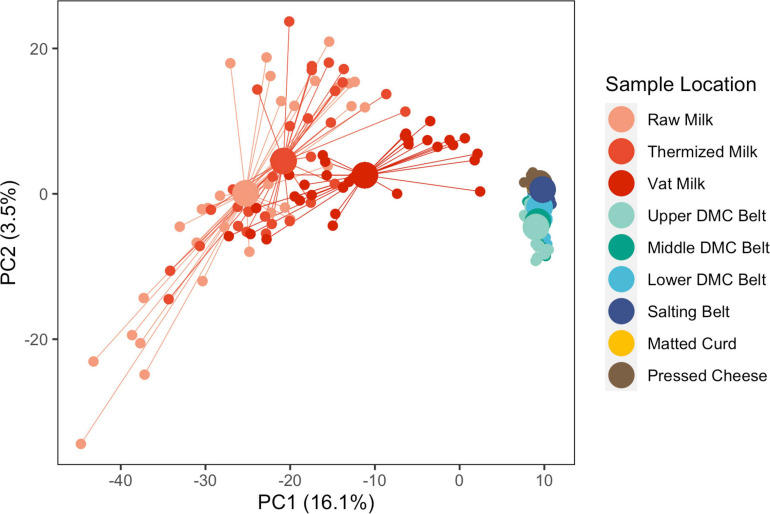
Principle component analysis of microbial communities from all sample locations and all facilities, following clr transformation. Samples are grouped by sample location. Individual samples (small points) are connected by segments to their corresponding group centroids (large points). Sequence variants which explained at least 5% of the component variance include *Clostridium* (2 SVs), Peptostreptococcaceae (1 SV), *Streptococcus* (2 SVs), and *Turicibacter* (1 SV) for PC1, and *Acinetobacter* (1 SV) and *Streptococcus* (1 SV) for PC2.

Beta diversity was evaluated at two levels: (i) between sample locations and (ii) within sample locations. Between sample locations, milk samples (raw, thermized, and vat milk) grouped distinctly from belt (DMC and salting belts) and cheese (matted curd and pressed cheese) samples across PC1, whereas belt and cheese samples were only weakly separated across PC2 (Figure 3). Milk samples exhibited greater within group variability (dispersion) than belt and cheese samples.

**FIGURE 4:**
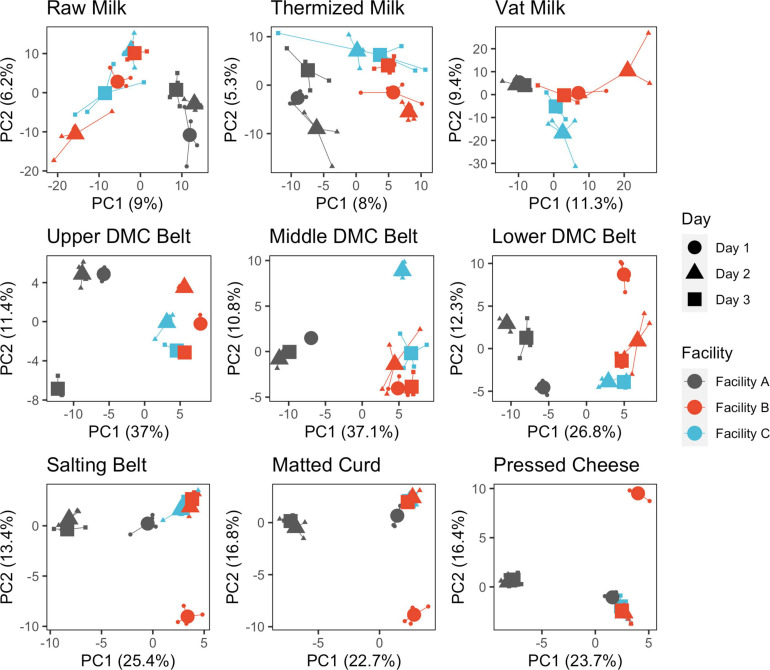
Principle component analysis of microbial communities following clr transformation and separated by sample location. Points are colored by facility and shaped by production day. Samples are grouped by production day nested within facility. Individual samples (small points) are connected by segments to their corresponding group centroids (large points).

Within sample locations, significant day-day and facility-facility differences were detected at each location (Figure 4, Supplementary Figure 3, and Table 2). Evidence of significant dispersion effects were also observed in many cases, though differences in group centroid locations for facility and production days were still clearly visible (Supplementary Table 2, Figure 4, and Supplementary Figure 3). The proportion of variance explained by the first two principle components was greatest for the belt swab samples, followed by cheese samples and then milk samples (Figure 4). Facility A was generally distinct from facilities B and C at each sample location, while facilities B and C became distinct only following thermization and were again mostly indistinguishable in the curd, salting belt, and cheese samples.

**TABLE 2:** Significance and explained variance of facility and production day nested within facility as determined by PERMANOVA of the Aitchison’s distance.

	Proportion of variance (*R*^2^)			Significance (*p*-value)	
Sample location	Facility	Day (Facility)	Residual	Facility	Day (Facility)
Raw milk	0.12	0.20	0.68	0.001	0.001
Thermized milk	0.11	0.19	0.70	0.001	0.001
Vat milk	0.16	0.18	0.66	0.001	0.003
Upper DMC belt	0.41	0.30	0.29	0.001	0.001
Middle DMC belt	0.43	0.21	0.35	0.001	0.001
Lower DMC belt	0.32	0.25	0.43	0.001	0.001
Salting belt	0.25	0.29	0.46	0.001	0.001
Matted curd	0.20	0.32	0.48	0.001	0.001
Pressed cheese	0.20	0.29	0.51	0.001	0.001

Regarding day-day differences, all production days formed distinct clusters within each facility in the milk and DMC belt samples but only formed distinct clusters on production day 1 within facilities A and B for the salting belt, matted curd, and pressed cheese (Figure 4). These last three sample locations exhibited similar grouping patterns and were attributed to daily starter rotations that occurred at each facility (Supplementary Figure 1). Within-day replicates were highly similar for belt and cheese samples but displayed moderate variability in the milk (Figure 4).

**FIGURE 5:**
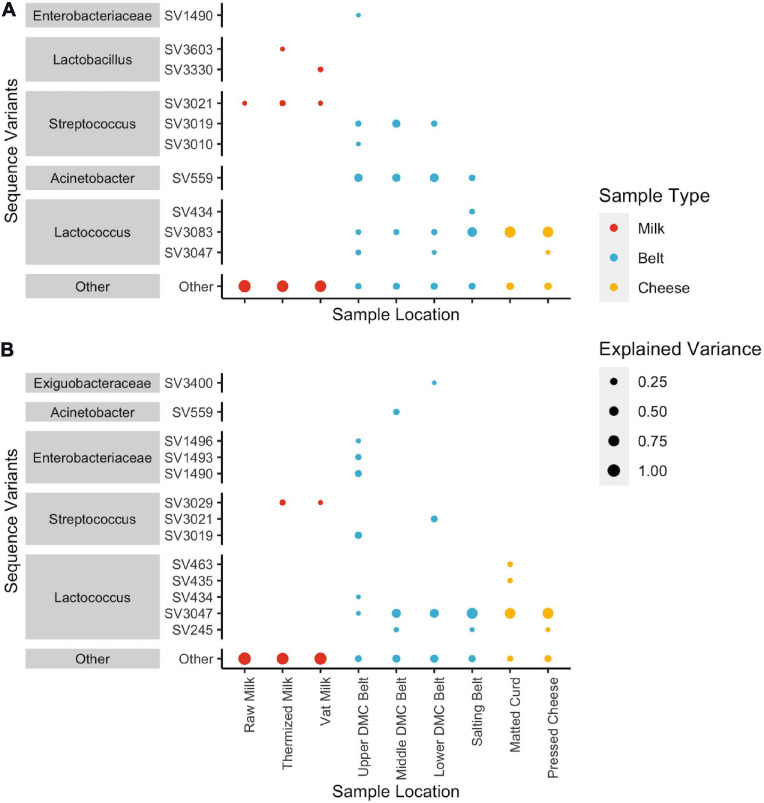
Dot plots showing squared loadings for **(A)** component 1 and **(B)** component 2 of the location-wise principle component analyses. Dot size represents the proportion of component variance explained by each SV. Dot color represent the general sample type (i.e., milk samples: raw, thermized, and vat milk; belt samples: DMC and salting belts; and cheese samples: matted curd and pressed cheese). SVs that explain < 5% of the component variance are grouped as “Other”.

**FIGURE 6:**
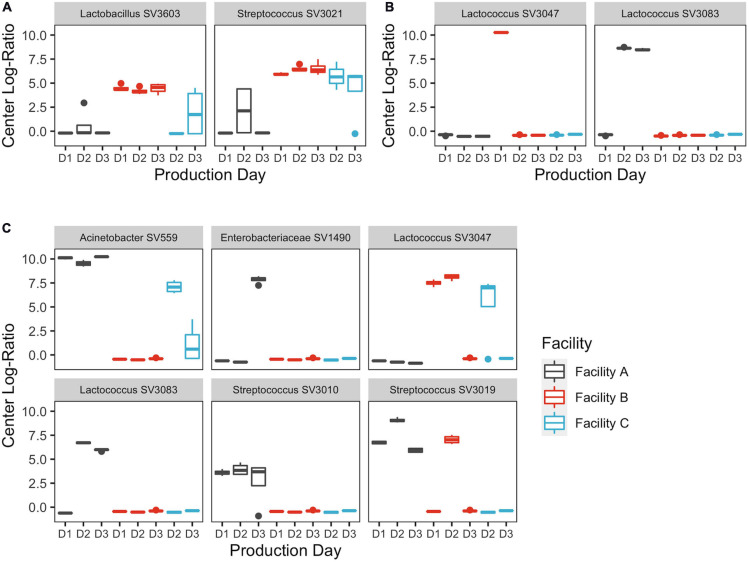
Centered log-ratios of SVs that explained ≥5% of the variance in the first component of the **(A)** thermized milk, **(B)** pressed cheese, and **(C)** upper DMC belt principle component analyses. Bars are colored by cheesemaking facility. Repeat sampling days within each facility are displayed across the *x*-axis.

SVs that commonly explained at least 5% of the variance in the first or second component of the within-location PCAs included *Streptococcus* and *Lactobacillus* for milk samples; *Lactococcus*, *Acinetobacter*, and *Streptococcus* for belt samples; and *Lactococcus* for cheese samples (Figure 5). Enterobacteriaceae and *Exiguobacterium* also contributed to >5% of the component variance for the upper and lower DMC belts, respectively. Further examination revealed that many of these SVs exhibited variations in their centered log-ratios according to facility and day of sampling (Figure 6). This agreed with the PERMANOVA models which found that both facility and production day nested within facility explained a large proportion of the community variance at each sample location (Table 2). Comparing these factors, facility explained a greater proportion of the variance in the DMC samples, whereas production day explained more variance in the remaining locations. Milk samples contained a large proportion of unexplained variance compared to the other sample types, again owing to their increased diversity.

### Changes in Relative Sequence Abundance and Bacterial Load

To gain greater insight into the factors that contributed to facility or daily differences in the alpha and beta diversity, changes in the relative abundance of starter and non-starter bacteria and their associated bacterial loads were assessed per sample location. The DMC bacterial community was also further evaluated by isolation and identification using16S rRNA sequencing.

**FIGURE 7:**
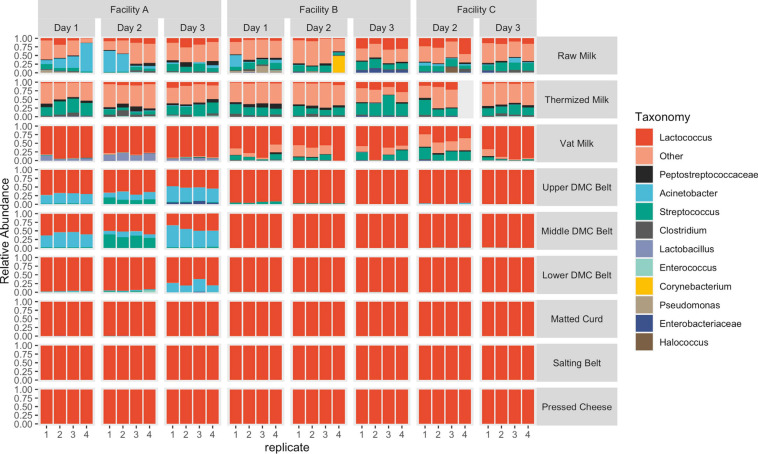
Relative abundance of taxonomic features separated by sample location. Features with a relative abundance >10% in the milk samples and >1% in the remaining locations were grouped as “Other.”

**FIGURE 8:**
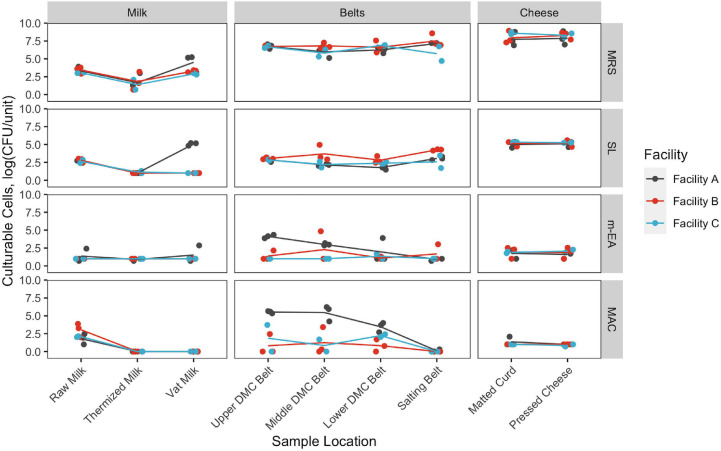
Changes in cell density throughout production. Cell density estimates are based on De Man, Rogosa and Sharpe agar (MRS), Rogosa SL agar (SL), m-Enterococcus agar (m-EA), and MacConkey agar (MAC). Density units vary by sample location: log CFU/mL for milk samples, log CFU/swab for belt samples, and log CFU/g for cheese samples. Samples are colored by facility. Points represent individual samples. Lines represent facility averages.

In agreement with beta-diversity analyses, clear differences in species richness/evenness and the relative abundance of dominant/subdominant taxa were observed between sample types, sample locations, facilities, and production days (Figure 7). Conversely, changes in bacterial load did not always reflect these differences (Figure 8). Milk samples were visibly more diverse than belt or cheese samples in terms of the number of unique colony morphologies observed on MRS (data not shown). Samples collected after the vat were typically dominated by *Lactococcus* sequences and had greater CFU counts of generic LAB, as determined on MRS agar (Figures 7, 8). CFU counts on Rogosa SL agar were comparably larger at Facility A in the vat milk and may be a consequence of residual starter lactobacilli remaining on the ladle used for collection at this facility (Table 1 and Figure 8). The decrease in relative abundance of Gammaproteobacteria and increase in Firmicutes following thermization (Figure 7) corresponded with a comparable decrease in presumptive coliforms (MAC agar) and increase in generic LAB cell densities (MRS agar) (Figure 8).

A resurgence of non-starter bacteria, including Gammaproteobacteria and *Streptococcus*, was observed in the DMC (Figures 7, 8), though the extent of which was not always reflected in the sequence data (Figure 7) and further differed by facility, production day, and the specific DMC belt (i.e., upper, middle, or lower) (Figures 7, 8). Most notable was the cell density observed on MAC agar in the DMC at Facility A, which exceeded 5.3, 4.2, and 2.7 log cfu/mL each day on the upper, middle, and lower DMC belts, respectively (Figure 8). Further investigation into the identities of bacterial isolates from the DMC revealed that *Acinetobacter* was the predominant genus on MAC agar, while *Streptococcus* was predominant on m-EA (Supplementary Table 3). Other genera observed at lower frequencies and/or lower cell densities included *Escherichia*, *Klebsiella*, *Pseudomonas*, and *Enterobacter* on MAC agar and *Enterococcus* on m-EA agar. Species and subspecies repeatedly isolated from the DMC included *Acinetobacter baumanii*, *Escherichia fergusonii*, *Enterococcus faecalis*, and *Streptococcus gallolyticus* subsp. *macedonicus* at Facility A; *Escherichia fergusoni* and *Escherichia* CP040443/*Escherichia* LFH at Facility B; and *Acinetobacter baumannii* and *Pseudomonas mosselii* at Facility C (Supplementary Table 3). Following the DMC, bacterial cell densities on m-EA and MAC were mostly below the detection limit (1 log CFU/mL), supporting observations that the *Lactococcus* starter dominated these samples (Figures 7, 8). Core SVs present in ≥ 90% of samples included *Lactococcus*, *Streptococcus*, *Clostridium*, *Turicibacter*, and Peptostreptococcaceae for milk samples; *Lactococcus* and *Enterococcus* for belt samples; and *Lactococcus* and *Lactobacillus* for cheese samples.

## Discussion

Few studies have attempted to characterize the temporal and facility-facility variability of microbial communities in the food production environment. A fundamental understanding of this variability is essential for answering questions related to the microbial ecology of food production systems and the consequences for food quality and safety. A primary goal of this research was to improve our understanding of microbial variability in the cheesemaking environment by quantifying facility-facility and day-day differences in the cheese facility microbiome.

### Microbial Diversity Throughout Cheddar Cheese Production

Production stage has been previously identified as a principle driver of community assembly in the cheese production environment (Bokulich and Mills, 2013; Falardeau et al., 2019) and likewise had a clear impact on microbial compositions in the present study. Production stages were defined as milk processing (raw, thermized, and vat milk), cheddaring (DMC swabs and matted curd), salting (salting belt swabs), and pressing (pressed cheese), and were further separated based on their sample type: milk, food contact surfaces, or cheese.

Microbial dynamics in milk are complex, as they are influenced by several factors including the farm and milk handling practices (Kable et al., 2016; Falardeau et al., 2019). As a result, microbial communities in the milk were highly diverse, leading to greater ambiguity in these samples, as evidenced by the large proportion of unexplained variance in the PERMANOVA models (66–70%). In contrast, microbial differences on food contact surfaces and in the cheese were comparably simple. Differences on the DMC belts were driven by large disparities in bacterial load and species richness, both of which were distinctly greater at Facility A, while cheese and salting belt samples were driven by differences in the composition and abundance of *Lactococcus* SVs and were an attributed consequence of starter addition which occurred in the vat.

The interpretation of microbial diversity in the cheesemaking environment was also dependent on the sample type. While both the matted curd and lower DMC belt swabs were collected from essentially the same location in the production environment, belt swabs generally had greater proportions of non-starter bacteria, particularly *Acinetobacter* at Facility A, whereas the matted curd was almost entirely dominated by *Lactococcus* SVs. These differences likely arose from two factors: (1) greater concentration of *Lactococcus* starter in the curd, as compared to the surrounding food contact environment, and (2) growth of non-starter bacteria on the lower DMC belt surface throughout the production day (Selover et al., 2021).

The co-inhabitance of starter and non-starter bacteria in the cheese production environment is regularly discussed (Stellato et al., 2015; Falardeau et al., 2019) and thus, it was not surprising to find *Lactococcus* starter at high abundance at all sample locations post-starter addition (i.e., those following thermization). However, an important consequence of this commingling of starter and non-starter bacteria was the obfuscation of low-level community members that could contribute importantly to cheese quality during the later production stages (i.e., ripening). This obfuscation was most apparent in the vat milk, where residual starter culture present in the milk-to-vat line resulted in a lower estimate of species richness as compared to the thermized milk, despite the same underlying community members being expected at both locations. Absence of samples collected prior to starter addition, these low-level microbial communities would likely go undetected and thus their industrial relevance may be overlooked. As such, future investigations into the cheese facility microbiome should consider taking samples both pre- and post-starter addition with the intention of capturing these underlying communities.

### Sources of Facility-Facility Microbial Diversity

Previous investigations into facility-facility differences in the cheese facility microbiome have relied on comparisons between facilities that were producing different cheese recipes on very different production equipment (Bokulich and Mills, 2013; Quijada et al., 2018). In the present study, facility differences were based on comparisons between facilities producing near-identical cheese recipes on near-identical processing equipment. This approach allowed for greater explanation of facility differences and their potential sources. One trend that was consistent throughout production was the distinction of Facility A from Facility B and Facility C. While several factors likely contributed to this distinction, some of the most apparent sources included the shared milk source at Facility B and Facility C, and the differences in age of equipment used at each facility.

Facilities B and C are neighboring facilities that share common milk sources from predominantly large-scale dry-lot dairy operations, whereas Facility A is approximately 250 miles (402 km) away and sources their milk from numerous smaller pasture-based dairy farms. As a result, microbial communities in the raw milk from facilities B and C shared greater similarities than those from Facility A. These differences were seemingly driven in large part by *Streptococcus* SV 3021, which was absent at Facility A, but accounted for up to 6.7 and 6.9% of the sequences at facilities B and C. This adds to the growing evidence that milk can exhibit farm-level microbial differences and also supports previous observations that streptococci are a primary contributor to farm-level differences (Kable et al., 2016; Skeie et al., 2019). Streptococci are among the most common bacteria isolated from the dairy environment and are notable for their roles in human health, animal health, and fermented dairy products (De Vuyst and Tsakalidou, 2008). Further investigation into the species identity of SV 3021 revealed it to most likely be *Streptococcus thermophilus*, which is associated with fermented dairy products, including yogurt and Swiss cheese, and has been used experimentally to produce low-fat Cheddar cheese (Broadbent et al., 2003; Iyer et al., 2010). While the impact of *Streptococcus* in Cheddar cheese production is understudied, further support of *Streptococcus* being a primary contributor to microbial variability in milk suggests it could be a source of quality variability in other dairy products, where its impact is better understood. The actual quality implications of these farm-level differences in the milk microbiome should be the focus of future studies.

Despite the shared milk source at facilities B and C, differences in their microbiota were evident following thermization. This was unexpected, given that each facility was following identical thermization protocols, and seems to suggest that subtle differences in processing equipment can result in noticeable changes in the facility microbiome. Among the SVs that contributed to differences in the thermized milk at facilities B and C was *Lactobacillus* SV 3603. Ratios of SV 3603 were similar in the raw milk at facilities B and C but became distinctly different following thermization. Lactobacilli play a central role in flavor and texture development in Cheddar cheese and are thus considered an important contributor to Cheddar cheese quality (Montel et al., 2014).

Facility differences in the DMC, while simpler in their presentation, were more difficult to attribute to any one factor and likely originated from several factors including the milk and the processing equipment. Indeed, the processing equipment used at each facility, while mostly the same in function and design, did contain subtle differences, particularly regarding their age. The DMC at Facility A has been in operation for approximately 10–15 years longer than those at facilities B and C, possibly explaining the increased bacterial load and species richness observed in the Facility A DMC. The likelihood of bacterial fouling increases with prolonged equipment use, in large part due to the roughening of food contact surfaces resulting from natural wear-and-tear (Van Houdt and Michiels, 2010; Selover et al., 2021). This hypothesis is supported by the observation that many bacterial species isolated from the DMC belts at Facility A are often associated with biofilm formation in dairy manufacturing environments (Cherif-Antar et al., 2016; Zou and Liu, 2018). These species included *Acinetobacter*, *Escherichia*, *Klebsiella*, and *Enterococcus*, many of which were repeatedly isolated on all 3 days of sampling.

### The Importance of Daily Changes in the Cheese Facility Microbiome

Evidence of widespread day-day variations in the cheese facility microbiome calls into question the importance of these daily changes for cheese product quality and their influence on the interpretation of spatial diversity. Many of the SVs that differed between facilities also differed between days within facilities. This included *Lactobacillus* SV 3603, which varied between facilities B and C in the thermized milk, along with others including *Streptococcus*, *Acinetobacter*, and Enterobacteriaceae. While all four bacteria are commonly isolated from the dairy environment, the quality implications of *Streptococcus* and *Acinetobacter* in Cheddar cheese are mostly unknown (Riquelme et al., 2015; Kable et al., 2016).

Detection of Enterobacteriaceae, which includes coliforms such as *Escherichia coli*, can be indicators of insanitary conditions in dairy manufacturing, in some cases leading to non-compliance with good manufacturing practices (Martin et al., 2016). The sudden increase in Enterobacteriaceae on the final day of sampling in the DMC at Facility A presents a possibly troubling scenario, where problematic bacteria may sporadically increase in cell density in the cheesemaking environment, thus leading to a corresponding increase in the cheese. This observation supports accounts described by the cheese manufacturer at Facility A, where coliforms are sporadically detected in the fresh cheese at low levels (Selover et al., 2021).

A popular belief for many fermented foods is that production facilities can harbor facility-specific microbial communities which impart unique “house” characteristics to the finished product. Evidence of the facility-specific microbiome has been based on observations that microbial communities can differ between facilities (Bokulich and Mills, 2013; Quijada et al., 2018). We argue that facility-facility differences do not necessarily imply specificity and that temporal variations in the facility microbiome may confound the interpretation of facility-facility variation when sampling efforts are insufficient. This argument is based on the recognition of significant day-day variability in the cheese facility microbiome and is supported by trends observed in the matted curd and pressed cheese.

Facility differences in the curd and cheese samples were attributed to an asynchronous rotation of starter blends, which occurred approximately every 24–48 h at each facility. While not all starter blends are shared between facilities, and therefore could be considered facility-specific, the facility-facility differences observed in the curd and cheese in the present study were driven by starter blends that are shared between facilities. Specifically, starter blend III, which was used at Facility A on days 1 and 2 but never at facilities B or C during the sampling period, appeared to be the primary driver of facility differences in the curd and cheese, despite this blend being common to all three facilities. It is expected that greater sampling efforts over a larger timespan would resolve this issue by showing that starter blend III is in fact common to each facility. Future investigations into the facility-specific microbiome should include larger timespans with the intent of differentiating temporal changes from facility changes, particularly those originating from the resident microbiome.

## Data Availability Statement

The datasets presented in this study can be found in online repositories. The names of the repository/repositories and accession number(s) can be found below: NCBI PRJNA687545.

## Author Contributions

JJ, CC, and JW-C conceived and designed the work. JJ collected the data, conducted data analysis and interpretation, and drafted the article. CC and JW-C critically revised the article. All authors contributed to the article and approved the submitted version.

## Conflict of Interest

The authors declare that the research was conducted in the absence of any commercial or financial relationships that could be construed as a potential conflict of interest.
