# Supplementary material for: The Cheese Production Facility Microbiome Exhibits Temporal and Spatial Variability
Source: Front Microbiol. 2021 Mar 9;12:644828. doi: 10.3389/fmicb.2021.644828 (PMC7985343; doi:10.3389/fmicb.2021.644828)
Supplement: Supplementary file 1 [file Data_Sheet_1.DOCX]

Supplementary Material


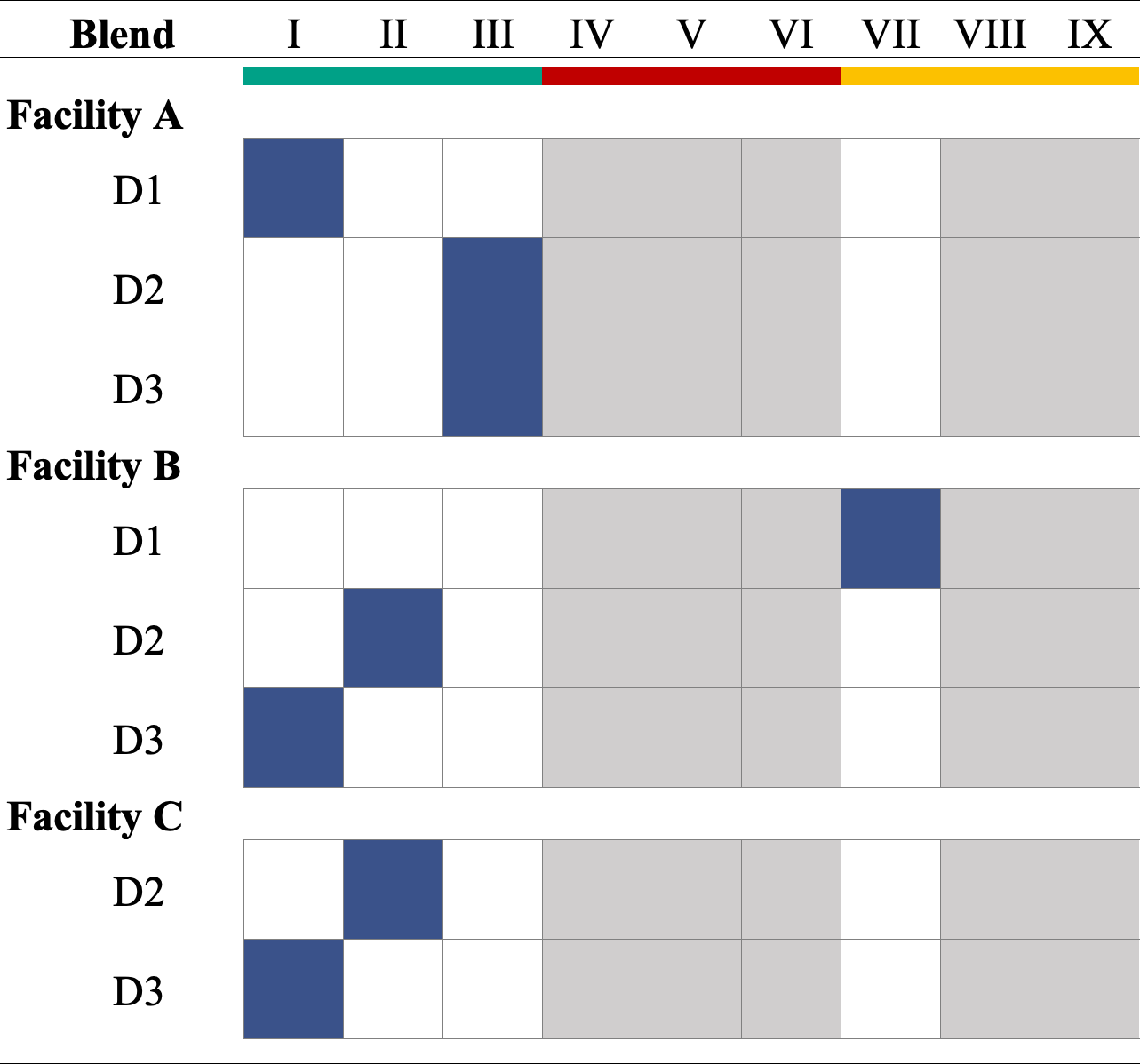


Supplementary Figure 1. Daily *Lactococcus* starter blends during the three-day sampling period. Starter blends are represented by columns. Starter strains are color coded by whether they are used at all three facilities (green), Facility B and Facility C only (yellow), or Facility A only (red). Blue squares denote when a starter blend was used. Gray squares denote starter blends that were not used during the sampling period.


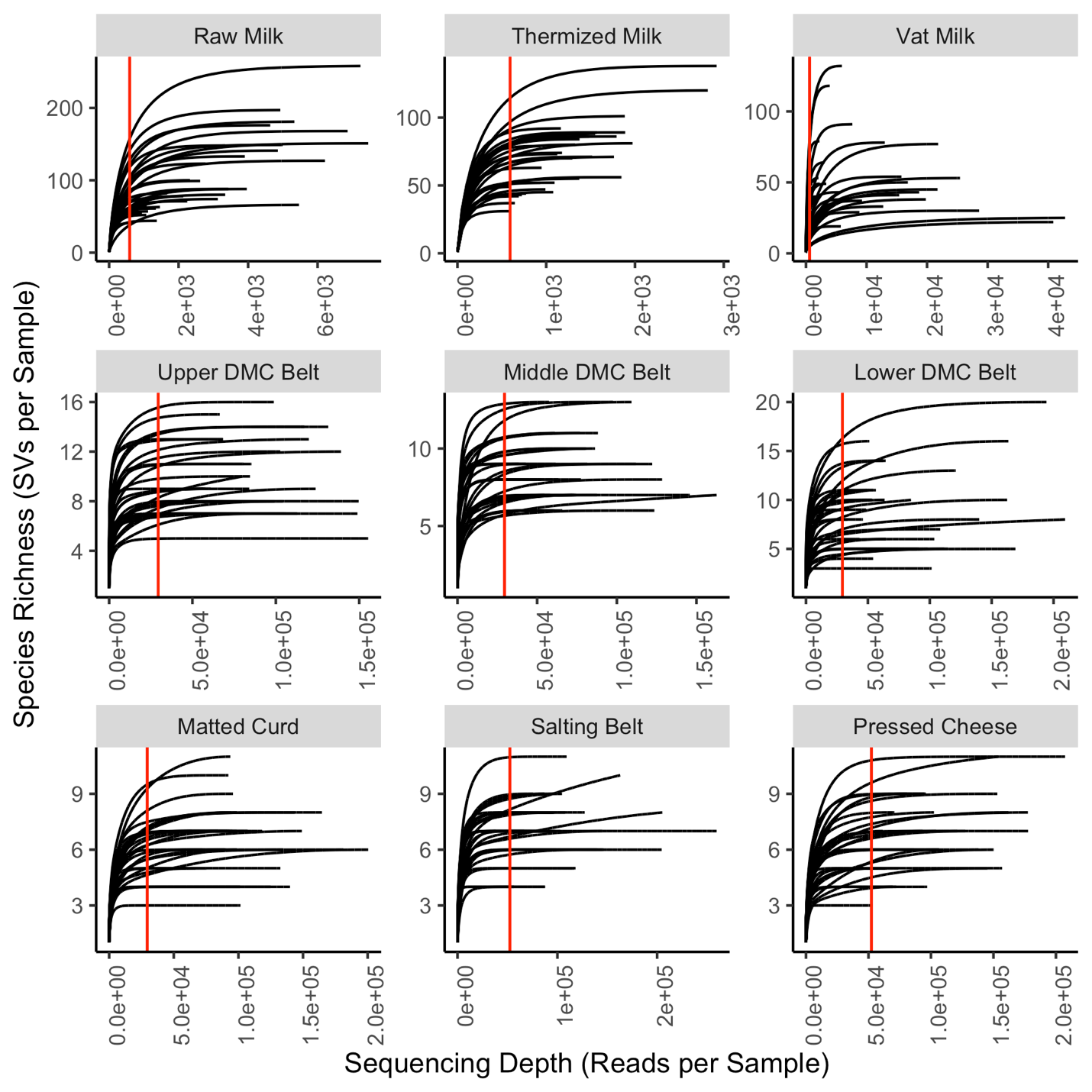


Supplemental Figure 2. Rarefaction curves of samples by sample location. Median depths following quality filtering and contaminant removal were 2220, 85122, and 96468 sequences per sample for milk, belt, and cheese samples. Given these disparities, samples were rarefied to different depths depending on their sample type (red vertical lines). Milk samples were rarefied to 593 sequences per sample, belt samples to 29467 sequences per sample, and cheese samples to 52372 sequences per sample. A single vat milk sample, which contained only 213 sequences, was not included in the diversity analysis.


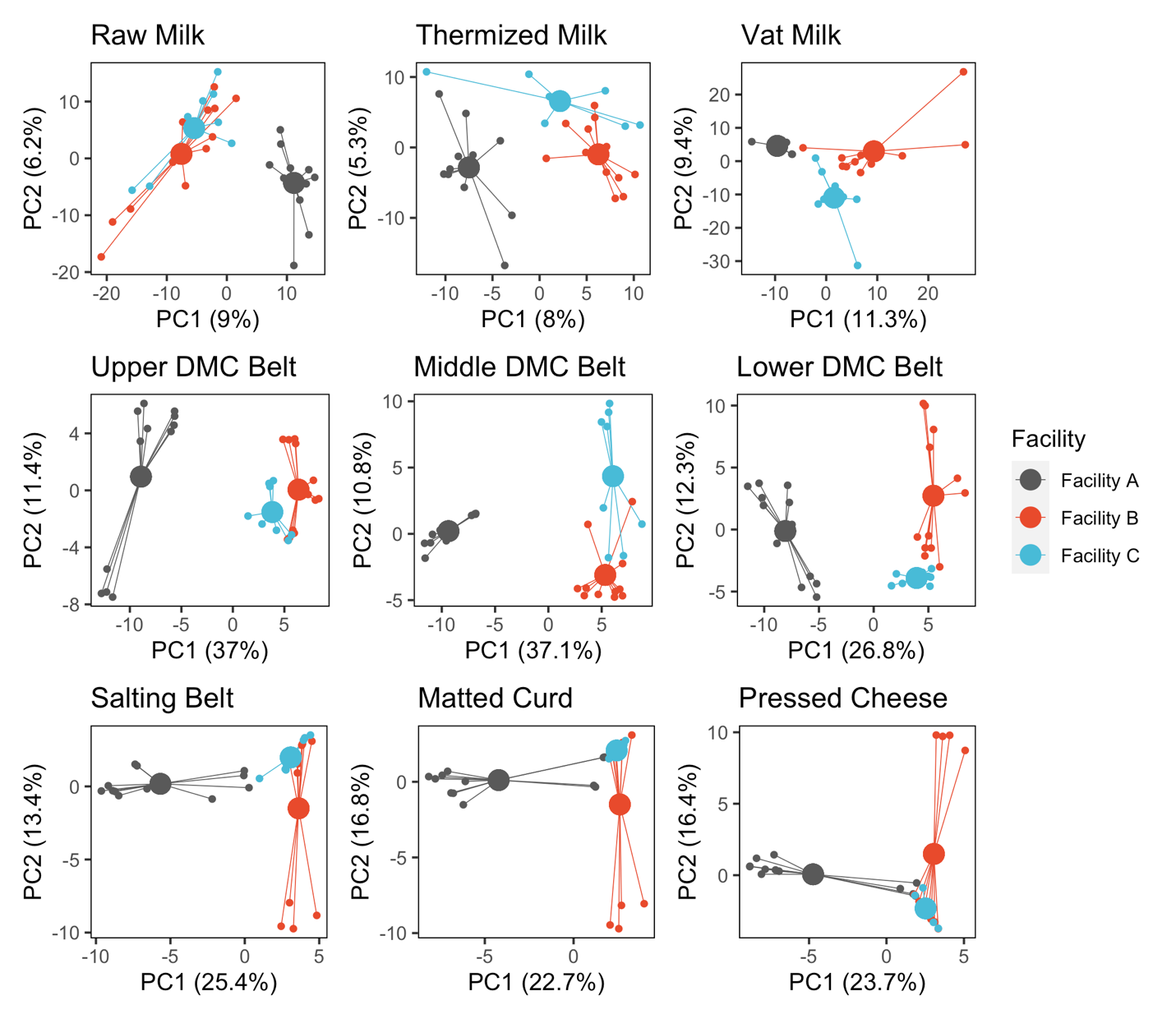


Supplemental Figure 3. Principle component analysis of microbial communities following clr transformation and separated by sample location. Points are grouped and color by facility. Individual samples (small points) are connected by segments to their corresponding group centroids (large points).

Supplemental Table 1. Primers used to generate dual-index libraries for 16S amplicon sequencing

| **Name-index** | **Primer type** | **Sequence** |
| --- | --- | --- |
| 515F-S502 | 16S forward | AATGATACGGCGACCACCGAGATCTACACCTCTCTATTCGTCGGCAGCGTCAGATGTGTATAAGAGACAGGTGYCAGCMGCCGCGGTAA |
| 515F-S503 | 16S forward | AATGATACGGCGACCACCGAGATCTACACTATCCTCTTCGTCGGCAGCGTCAGATGTGTATAAGAGACAGGTGYCAGCMGCCGCGGTAA |
| 515F-S505 | 16S forward | AATGATACGGCGACCACCGAGATCTACACGTAAGGAGTCGTCGGCAGCGTCAGATGTGTATAAGAGACAGGTGYCAGCMGCCGCGGTAA |
| 515F-S506 | 16S forward | AATGATACGGCGACCACCGAGATCTACACACTGCATATCGTCGGCAGCGTCAGATGTGTATAAGAGACAGGTGYCAGCMGCCGCGGTAA |
| 515F-S507 | 16S forward | AATGATACGGCGACCACCGAGATCTACACAAGGAGTATCGTCGGCAGCGTCAGATGTGTATAAGAGACAGGTGYCAGCMGCCGCGGTAA |
| 515F-S508 | 16S forward | AATGATACGGCGACCACCGAGATCTACACCTAAGCCTTCGTCGGCAGCGTCAGATGTGTATAAGAGACAGGTGYCAGCMGCCGCGGTAA |
| 515F-S510 | 16S forward | AATGATACGGCGACCACCGAGATCTACACCGTCTAATTCGTCGGCAGCGTCAGATGTGTATAAGAGACAGGTGYCAGCMGCCGCGGTAA |
| 515F-S511 | 16S forward | AATGATACGGCGACCACCGAGATCTACACTCTCTCCGTCGTCGGCAGCGTCAGATGTGTATAAGAGACAGGTGYCAGCMGCCGCGGTAA |
| 515F-S513 | 16S forward | AATGATACGGCGACCACCGAGATCTACACTCGACTAGTCGTCGGCAGCGTCAGATGTGTATAAGAGACAGGTGYCAGCMGCCGCGGTAA |
| 515F-S515 | 16S forward | AATGATACGGCGACCACCGAGATCTACACTTCTAGCTTCGTCGGCAGCGTCAGATGTGTATAAGAGACAGGTGYCAGCMGCCGCGGTAA |
| 515F-S516 | 16S forward | AATGATACGGCGACCACCGAGATCTACACCCTAGAGTTCGTCGGCAGCGTCAGATGTGTATAAGAGACAGGTGYCAGCMGCCGCGGTAA |
| 515F-S517 | 16S forward | AATGATACGGCGACCACCGAGATCTACACGCGTAAGATCGTCGGCAGCGTCAGATGTGTATAAGAGACAGGTGYCAGCMGCCGCGGTAA |
| 515F-S518 | 16S forward | AATGATACGGCGACCACCGAGATCTACACCTATTAAGTCGTCGGCAGCGTCAGATGTGTATAAGAGACAGGTGYCAGCMGCCGCGGTAA |
| 515F-S520 | 16S forward | AATGATACGGCGACCACCGAGATCTACACAAGGCTATTCGTCGGCAGCGTCAGATGTGTATAAGAGACAGGTGYCAGCMGCCGCGGTAA |
| 515F-S521 | 16S forward | AATGATACGGCGACCACCGAGATCTACACGAGCCTTATCGTCGGCAGCGTCAGATGTGTATAAGAGACAGGTGYCAGCMGCCGCGGTAA |
| 515F-S522 | 16S forward | AATGATACGGCGACCACCGAGATCTACACTTATGCGATCGTCGGCAGCGTCAGATGTGTATAAGAGACAGGTGYCAGCMGCCGCGGTAA |
| 926R-N701 | 16S reverse | CAAGCAGAAGACGGCATACGAGATTCGCCTTAGTCTCGTGGGCTCGGAGATGTGTATAAGAGACAGCCGYCAATTYMTTTRAGTTT |
| 926R-N702 | 16S reverse | CAAGCAGAAGACGGCATACGAGATCTAGTACGGTCTCGTGGGCTCGGAGATGTGTATAAGAGACAGCCGYCAATTYMTTTRAGTTT |
| 926R-N703 | 16S reverse | CAAGCAGAAGACGGCATACGAGATTTCTGCCTGTCTCGTGGGCTCGGAGATGTGTATAAGAGACAGCCGYCAATTYMTTTRAGTTT |
| 926R-N704 | 16S reverse | CAAGCAGAAGACGGCATACGAGATGCTCAGGAGTCTCGTGGGCTCGGAGATGTGTATAAGAGACAGCCGYCAATTYMTTTRAGTTT |
| 926R-N705 | 16S reverse | CAAGCAGAAGACGGCATACGAGATAGGAGTCCGTCTCGTGGGCTCGGAGATGTGTATAAGAGACAGCCGYCAATTYMTTTRAGTTT |
| 926R-N706 | 16S reverse | CAAGCAGAAGACGGCATACGAGATCATGCCTAGTCTCGTGGGCTCGGAGATGTGTATAAGAGACAGCCGYCAATTYMTTTRAGTTT |
| 926R-N707 | 16S reverse | CAAGCAGAAGACGGCATACGAGATGTAGAGAGGTCTCGTGGGCTCGGAGATGTGTATAAGAGACAGCCGYCAATTYMTTTRAGTTT |
| 926R-N710 | 16S reverse | CAAGCAGAAGACGGCATACGAGATCAGCCTCGGTCTCGTGGGCTCGGAGATGTGTATAAGAGACAGCCGYCAATTYMTTTRAGTTT |
| 926R-N711 | 16S reverse | CAAGCAGAAGACGGCATACGAGATTGCCTCTTGTCTCGTGGGCTCGGAGATGTGTATAAGAGACAGCCGYCAATTYMTTTRAGTTT |
| 926R-N712 | 16S reverse | CAAGCAGAAGACGGCATACGAGATTCCTCTACGTCTCGTGGGCTCGGAGATGTGTATAAGAGACAGCCGYCAATTYMTTTRAGTTT |
| 926R-N714 | 16S reverse | CAAGCAGAAGACGGCATACGAGATTCATGAGCGTCTCGTGGGCTCGGAGATGTGTATAAGAGACAGCCGYCAATTYMTTTRAGTTT |
| 926R-N715 | 16S reverse | CAAGCAGAAGACGGCATACGAGATCCTGAGATGTCTCGTGGGCTCGGAGATGTGTATAAGAGACAGCCGYCAATTYMTTTRAGTTT |
| 926R-N716 | 16S reverse | CAAGCAGAAGACGGCATACGAGATTAGCGAGTGTCTCGTGGGCTCGGAGATGTGTATAAGAGACAGCCGYCAATTYMTTTRAGTTT |
| 926R-N718 | 16S reverse | CAAGCAGAAGACGGCATACGAGATGTAGCTCCGTCTCGTGGGCTCGGAGATGTGTATAAGAGACAGCCGYCAATTYMTTTRAGTTT |
| 926R-N719 | 16S reverse | CAAGCAGAAGACGGCATACGAGATTACTACGCGTCTCGTGGGCTCGGAGATGTGTATAAGAGACAGCCGYCAATTYMTTTRAGTTT |
| 926R-N720 | 16S reverse | CAAGCAGAAGACGGCATACGAGATAGGCTCCGGTCTCGTGGGCTCGGAGATGTGTATAAGAGACAGCCGYCAATTYMTTTRAGTTT |
| 926R-N721 | 16S reverse | CAAGCAGAAGACGGCATACGAGATGCAGCGTAGTCTCGTGGGCTCGGAGATGTGTATAAGAGACAGCCGYCAATTYMTTTRAGTTT |
| 926R-N722 | 16S reverse | CAAGCAGAAGACGGCATACGAGATCTGCGCATGTCTCGTGGGCTCGGAGATGTGTATAAGAGACAGCCGYCAATTYMTTTRAGTTT |
| 926R-N723 | 16S reverse | CAAGCAGAAGACGGCATACGAGATGAGCGCTAGTCTCGTGGGCTCGGAGATGTGTATAAGAGACAGCCGYCAATTYMTTTRAGTTT |
| 926R-N724 | 16S reverse | CAAGCAGAAGACGGCATACGAGATCGCTCAGTGTCTCGTGGGCTCGGAGATGTGTATAAGAGACAGCCGYCAATTYMTTTRAGTTT |
| 926R-N726 | 16S reverse | CAAGCAGAAGACGGCATACGAGATGTCTTAGGGTCTCGTGGGCTCGGAGATGTGTATAAGAGACAGCCGYCAATTYMTTTRAGTTT |
| 926R-N727 | 16S reverse | CAAGCAGAAGACGGCATACGAGATACTGATCGGTCTCGTGGGCTCGGAGATGTGTATAAGAGACAGCCGYCAATTYMTTTRAGTTT |
| 926R-N728 | 16S reverse | CAAGCAGAAGACGGCATACGAGATTAGCTGCAGTCTCGTGGGCTCGGAGATGTGTATAAGAGACAGCCGYCAATTYMTTTRAGTTT |
| 926R-N729 | 16S reverse | CAAGCAGAAGACGGCATACGAGATGACGTCGAGTCTCGTGGGCTCGGAGATGTGTATAAGAGACAGCCGYCAATTYMTTTRAGTTT |

Supplemental Table 2. PERMDISP results

| **Sample Location** | **Facility** | **Day (Facility A)** | **Day (Facility B)** | **Day (Facility C)** |
| --- | --- | --- | --- | --- |
| Raw Milk | ns | ns | <0.001 | ns |
| Thermized Milk | ns | ns | 0.007 | ns |
| Vat Milk | <0.001 | ns | ns | 0.019 |
| Upper DMC Belt | 0.006 | ns | ns | ns |
| Middle DMC Belt | ns | ns | ns | ns |
| Lower DMC Belt | <0.001 | 0.021 | 0.037 | ns |
| Salting Belt | 0.034 | ns | ns | ns |
| Matted Curd | 0.005 | ns | ns | ns |
| Pressed Cheese | <0.001 | ns | ns | ns |

Supplemental Table 3. 16S rRNA taxonomic identities of bacteria isolated from MAC and m-EA agars from the DMC belts.

| **Isolate ID** | **Media** | **Facility** | **Day** | **Location** | **Top Hit(s)** | **Similarity** | **Accession(s)** |
| --- | --- | --- | --- | --- | --- | --- | --- |
| E18 | m-EA | Facility A | Day 1 | Upper DMC Belt | *Enterococcus faecalis* | 100% | [ASDA01000001](https://www.ezbiocloud.net/16SrRNA?ac=ASDA01000001) |
| E20 | m-EA | Facility A | Day 1 | Upper DMC Belt | *Streptococcus gallolyticus* subsp. *macedonicus* | 100% | [UHFM01000006](https://www.ezbiocloud.net/16SrRNA?ac=UHFM01000006) |
| E36 | m-EA | Facility A | Day 2 | Upper DMC Belt | *Enterococcus faecalis* | 99.71% | [ASDA01000001](https://www.ezbiocloud.net/16SrRNA?ac=ASDA01000001) |
| E38 | m-EA | Facility A | Day 2 | Upper DMC Belt | *Streptococcus gallolyticus* subsp. *macedonicus* | 99.39% | [UHFM01000006](https://www.ezbiocloud.net/16SrRNA?ac=UHFM01000006) |
| E40 | m-EA | Facility A | Day 2 | Middle DMC Belt | *Enterococcus faecalis* | 100% | [ASDA01000001](https://www.ezbiocloud.net/16SrRNA?ac=ASDA01000001) |
| E42 | m-EA | Facility A | Day 2 | Middle DMC Belt | *Streptococcus gallolyticus* subsp. *macedonicus* | 100% | [UHFM01000006](https://www.ezbiocloud.net/16SrRNA?ac=UHFM01000006) |
| E58 | m-EA | Facility A | Day 3 | Upper DMC Belt | *Enterococcus faecalis* | 94.54% | [ASDA01000001](https://www.ezbiocloud.net/16SrRNA?ac=ASDA01000001) |
| E59 | m-EA | Facility A | Day 3 | Upper DMC Belt | *Streptococcus gallolyticus* subsp. *macedonicus* | 99.49% | [UHFM01000006](https://www.ezbiocloud.net/16SrRNA?ac=UHFM01000006) |
| E62 | m-EA | Facility A | Day 3 | Middle DMC Belt | *Enterococcus faecalis* | 91.01% | [ASDA01000001](https://www.ezbiocloud.net/16SrRNA?ac=ASDA01000001) |
| E64 | m-EA | Facility A | Day 3 | Middle DMC Belt | *Streptococcus gallolyticus* subsp. *macedonicus* | 100% | [UHFM01000006](https://www.ezbiocloud.net/16SrRNA?ac=UHFM01000006) |
| E70 | m-EA | Facility A | Day 3 | Upper DMC Belt | *Streptococcus gallolyticus* subsp. *macedonicus* | 99.78% | [UHFM01000006](https://www.ezbiocloud.net/16SrRNA?ac=UHFM01000006) |
| E71 | m-EA | Facility A | Day 3 | Lower DMC Belt | *Streptococcus gallolyticus* subsp. *macedonicus* | 99.49% | [UHFM01000006](https://www.ezbiocloud.net/16SrRNA?ac=UHFM01000006) |
| E77 | m-EA | Facility B | Day 1 | Upper DMC Belt | *Streptococcus gallolyticus* subsp. *macedonicus* | 99.78% | [UHFM01000006](https://www.ezbiocloud.net/16SrRNA?ac=UHFM01000006) |
| E79 | m-EA | Facility B | Day 1 | Lower DMC Belt | *Enterococcus durans* | 99.33% | [BCQB01000108](https://www.ezbiocloud.net/16SrRNA?ac=BCQB01000108) |
| E91 | m-EA | Facility B | Day 3 | Middle DMC Belt | *Enterococcus faecalis* | 99.86% | [ASDA01000001](https://www.ezbiocloud.net/16SrRNA?ac=ASDA01000001) |
| E120 | m-EA | Facility B | Day 3 | Lower DMC Belt | *Enterococcus faecalis* | 100% | [ASDA01000001](https://www.ezbiocloud.net/16SrRNA?ac=ASDA01000001) |
| E106 | m-EA | Facility C | Day 2 | Lower DMC Belt | *Enterococcus faecalis* | 99.86% | [ASDA01000001](https://www.ezbiocloud.net/16SrRNA?ac=ASDA01000001) |
| C10 | MAC | Facility A | Day 1 | Upper DMC Belt | *Acinetobacter baumannii* | 100% | [ACQB01000091](https://www.ezbiocloud.net/16SrRNA?ac=ACQB01000091) |
| C12 | MAC | Facility A | Day 1 | Upper DMC Belt | *Escherichia fergusonii* | 100% | [CU928158](https://www.ezbiocloud.net/16SrRNA?ac=CU928158) |
| C13 | MAC | Facility A | Day 1 | Middle DMC Belt | *Acinetobacter baumannii* | 99.78% | [ACQB01000091](https://www.ezbiocloud.net/16SrRNA?ac=ACQB01000091) |
| C14 | MAC | Facility A | Day 1 | Middle DMC Belt | *Shigella flexneri* / *Escherichia fergusonii* / *Escherichia coli* | 100% | [X96963 / CU928158 / X80725](https://www.ezbiocloud.net/16SrRNA?ac=X96963) |
| C16 | MAC | Facility A | Day 1 | Middle DMC Belt | *Acinetobacter baumannii* | 99.72% | [ACQB01000091](https://www.ezbiocloud.net/16SrRNA?ac=ACQB01000091) |
| C17 | MAC | Facility A | Day 1 | Lower DMC Belt | *Acinetobacter baumannii* | 99.93% | [ACQB01000091](https://www.ezbiocloud.net/16SrRNA?ac=ACQB01000091) |
| C31 | MAC | Facility A | Day 2 | Middle DMC Belt | *Shigella flexneri* / *Escherichia fergusonii* | 99.81% | [X96963 / CU928158](https://www.ezbiocloud.net/16SrRNA?ac=X96963) |
| C33 | MAC | Facility A | Day 2 | Upper DMC Belt | *Klebsiella variicola* subsp. *variicola* | 99.14% | [CP010523](https://www.ezbiocloud.net/16SrRNA?ac=CP010523) |
| C36 | MAC | Facility A | Day 2 | Upper DMC Belt | *Shigella flexneri* / *Escherichia fergusonii* | 99.90% | [X96963 / CU928158](https://www.ezbiocloud.net/16SrRNA?ac=X96963) |
| C38 | MAC | Facility A | Day 2 | Middle DMC Belt | *Acinetobacter baumannii* | 100% | [ACQB01000091](https://www.ezbiocloud.net/16SrRNA?ac=ACQB01000091) |
| C41 | MAC | Facility A | Day 2 | Lower DMC Belt | *Acinetobacter baumannii* | 100% | [ACQB01000091](https://www.ezbiocloud.net/16SrRNA?ac=ACQB01000091) |
| C42 | MAC | Facility A | Day 2 | Lower DMC Belt | *Acinetobacter baumannii* | 99.90% | [ACQB01000091](https://www.ezbiocloud.net/16SrRNA?ac=ACQB01000091) |
| C61 | MAC | Facility A | Day 3 | Upper DMC Belt | *Klebsiella quasivariicola* | 99.78% | [CP022823](https://www.ezbiocloud.net/16SrRNA?ac=CP022823) |
| C64 | MAC | Facility A | Day 3 | Upper DMC Belt | *Acinetobacter baumannii* | 99.80% | [ACQB01000091](https://www.ezbiocloud.net/16SrRNA?ac=ACQB01000091) |
| C65 | MAC | Facility A | Day 3 | Upper DMC Belt | *Klebsiella pneumoniae* subsp. *ozaenae* | 95.15% | [Y17654](https://www.ezbiocloud.net/16SrRNA?ac=Y17654) |
| C67 | MAC | Facility A | Day 3 | Middle DMC Belt | *Acinetobacter baumannii* | 100% | [ACQB01000091](https://www.ezbiocloud.net/16SrRNA?ac=ACQB01000091) |
| C68 | MAC | Facility A | Day 3 | Middle DMC Belt | *Acinetobacter baumannii* | 99.81% | [ACQB01000091](https://www.ezbiocloud.net/16SrRNA?ac=ACQB01000091) |
| C69 | MAC | Facility A | Day 3 | Lower DMC Belt | *Shigella flexneri* / *Escherichia fergusonii* | 100% | [X96963 / CU928158](https://www.ezbiocloud.net/16SrRNA?ac=X96963) |
| C71 | MAC | Facility A | Day 3 | Lower DMC Belt | *Acinetobacter baumannii* | 100% | [ACQB01000091](https://www.ezbiocloud.net/16SrRNA?ac=ACQB01000091) |
| C74 | MAC | Facility A | Day 3 | Lower DMC Belt | *Klebsiella quasivariicola* / *Klebsiella variicola* subsp. *variicola* | 99.70% | [CP022823 / CP010523](https://www.ezbiocloud.net/16SrRNA?ac=CP022823) |
| C78 | MAC | Facility B | Day 1 | Lower DMC Belt | *Escherichia* LFHY | 99.32% | [LFHY01000009](https://www.ezbiocloud.net/16SrRNA?ac=LFHY01000009) |
| C80 | MAC | Facility B | Day 1 | Lower DMC Belt | *Escherichia* CP040443 / *Escherichia* LFHY | 99.90% | [CP040443 / LFHY01000009](https://www.ezbiocloud.net/16SrRNA?ac=CP040443) |
| C81 | MAC | Facility B | Day 2 | Lower DMC Belt | *Shigella flexneri* / *Escherichia fergusonii* | 100% | [X96963 / CU928158](https://www.ezbiocloud.net/16SrRNA?ac=X96963) |
| C83 | MAC | Facility B | Day 3 | Upper DMC Belt | *Escherichia* CP040443 / *Escherichia* LFHY | 99.60% | [CP040443 / LFHY01000009](https://www.ezbiocloud.net/16SrRNA?ac=CP040443) |
| C84 | MAC | Facility B | Day 3 | Middle DMC Belt | *Escherichia fergusonii* | 100% | [CU928158](https://www.ezbiocloud.net/16SrRNA?ac=CU928158) |
| C85 | MAC | Facility B | Day 3 | Middle DMC Belt | *Acinetobacter baumannii* | 99.28% | [ACQB01000091](https://www.ezbiocloud.net/16SrRNA?ac=ACQB01000091) |
| C87 | MAC | Facility B | Day 3 | Middle DMC Belt | *Shigella flexneri* / *Escherichia ferhusonii* | 99.47% | [X96963 / CU928158](https://www.ezbiocloud.net/16SrRNA?ac=X96963) |
| C88 | MAC | Facility B | Day 3 | Lower DMC Belt | *Enterobacter* spp. | 99.59% | [FYBA01000020 / FYBI01000003 / FYBF01000083 / MK567958 / CP034769](https://www.ezbiocloud.net/16SrRNA?ac=FYBA01000020) |
| C92 | MAC | Facility C | Day 2 | Upper DMC Belt | *Acinetobacter baumannii* | 100% | [ACQB01000091](https://www.ezbiocloud.net/16SrRNA?ac=ACQB01000091) |
| C97 | MAC | Facility C | Day 2 | Lower DMC Belt | *Pseudomonas* spp. | 99.34% | [AF072688 / QKVM01000121 / FMYX01000029 / CP016634](https://www.ezbiocloud.net/16SrRNA?ac=AF072688) |
| C98 | MAC | Facility C | Day 2 | Lower DMC Belt | *Pseudomonas mosselii* | 99.85% | [AF072688](https://www.ezbiocloud.net/16SrRNA?ac=AF072688) |
| C102 | MAC | Facility C | Day 3 | Upper DMC Belt | *Acinetobacter baumannii* | 100% | [ACQB01000091](https://www.ezbiocloud.net/16SrRNA?ac=ACQB01000091) |
| C103 | MAC | Facility C | Day 3 | Upper DMC Belt | *Escherichia* LFHY | 100% | [LFHY01000009](https://www.ezbiocloud.net/16SrRNA?ac=LFHY01000009) |
| C107 | MAC | Facility C | Day 3 | Lower DMC Belt | *Acinetobacter baumannii* | 98.15% | [ACQB01000091](https://www.ezbiocloud.net/16SrRNA?ac=ACQB01000091) |
